# Multimodal Emotion Recognition from Art Using Sequential Co-Attention

**DOI:** 10.3390/jimaging7080157

**Published:** 2021-08-21

**Authors:** Tsegaye Misikir Tashu, Sakina Hajiyeva, Tomas Horvath

**Affiliations:** 1 Department of Data Science and Engineering (T-Labs), Faculty of Informatics, Eötvös Loránd University, Pázmány Péter Sétány 1/C, 1117 Budapest, Hungary; Hajieva44@gmail.com (S.H.); tomas.horvath@inf.elte.hu (T.H.); 2 College of Informatics, Kombolcha Institute of Technology, Wollo University, Kombolcha 208, Ethiopia; 3 Faculty of Science Institute of Computer Science, Pavol Jozef Šafárik University, Jesenná 5, 040 01 Košice, Slovakia

**Keywords:** multimodal, emotions, attention, art, modality fusion, emotion analysis

## Abstract

In this study, we present a multimodal emotion recognition architecture that uses both feature-level attention (sequential co-attention) and modality attention (weighted modality fusion) to classify emotion in art. The proposed architecture helps the model to focus on learning informative and refined representations for both feature extraction and modality fusion. The resulting system can be used to categorize artworks according to the emotions they evoke; recommend paintings that accentuate or balance a particular mood; search for paintings of a particular style or genre that represents custom content in a custom state of impact. Experimental results on the WikiArt emotion dataset showed the efficiency of the approach proposed and the usefulness of three modalities in emotion recognition.

## 1. Introduction

Art is an imaginative human creation that should be appreciated, make people think, and evoke an emotional response [1,2,3]. Emotion is a psycho-physiological process that can be triggered by conscious and/or unconscious perceptions of objects and situations and is related to a variety of factors such as mood, temperament, personality, disposition, and motivation [2,3]. Emotions are very important in human decision making, interaction and cognitive processes [4]. As technology advances and our understanding of emotions grows, so does the need for automatic emotion recognition systems [2]. Automatic emotion recognition has been used for various applications including human–computer interactions [5], surveillance [6], robotics, gaming, entertainment, and more.

Initial work on emotion recognition was mostly carried out using unimodal [7,8] approaches. Unimodal modality can correspond to facial expressions, voice, text, posture, gaits, or physiological signals. This was followed by multimodal emotion recognition [9,10], where different modalities were used and combined in various ways to derive emotions.

However, most of the work on automatic analysis of artworks has focused on inferring painting styles [11], investigating influences between artists and art movements [12], distinguishing authentic drawings from imitations, automatically generating artworks [13], and evaluating evoked emotions [14,15]. There are also attempts to develop approaches to analyze people’s emotional experiences in response to artworks [14,15]. Most of these studies use computer vision and machine learning approaches to emotionally categorize artworks [14,15] and identify the parts of paintings that are responsible for evoking certain emotions [16].

Automatic detection of emotions evoked by art paintings is of significant importance as the results can be used to group art paintings according to the emotions they evoke, to provide painting recommendations that accentuate or balance a particular mood, and to find art paintings of a particular style or genre that represent user-defined content in a user-defined state of effect [1,2,3].

We proposed a co-attention-based multimodal emotion recognition model that jointly identifies reasons from all modalities used and a weighted modality fusion that provides feature-level system fusion and applies weighted modality scores over the extracted features to indicate the importance of the different modalities. We compared our approach to several baseline methods by testing the performance on the WikiArt emotion dataset [1], a benchmark dataset for emotion recognition in art. Our models can be used if the two modalities, namely the image (painting) and title (textual description), are provided. The third modality which is the emotion category is not possible to collect every time the model is used as its values come from the expert judgments. As the model was trained using the three modalities and to avoid any bugs during the deployment due to the missing category modality, we included a function that initializes the category modality into some value drawn randomly from a uniform distribution when the category modality is not present. The contribution of this paper can be summarized as follows:

1. We proposed a co-attention-based multimodal emotion recognition approach that aims to use information from the painting, title, and emotion category channels via weighted fusion to achieve more robust and accurate recognition;
2. An experiment was carried on the dataset collected and provided for emotion recognition, which is publicly available;
3. The proposed approach result was compared with the latest state-of-the-art approaches and also with other baseline approaches based on deep learning methods.

The rest of the paper is organized into five sections. Section 2 describes related works that are relevant to our research. Section 3 presents the proposed sequential multimodal fusion model architecture and Section 4 presents the overall experimental settings, implementation, and evaluation of the proposed system and results. Finally, Section 5 presents the conclusion.

## 2. Related Work

Emotion detection and sentiment analysis has been an area of interest for many decades and has always attracted attention in multiple fields using computer vision and natural language processing techniques. Depending on the number (uni- and multimodal) and types of modalities (speech, text, video, image), there have been some major improvements in the topic of emotion detection and sentiment analysis. In this section, we will focus on the most recent findings for unimodal and multimodal emotion recognition by discussing recent developments in techniques and approaches for each modality type.

### 2.1. Unimodal Approaches

The first attempts to identify human emotions were mostly unimodal. The most commonly studied modalities are facial expressions [7], speech or vocal expressions [17], body gestures [18], and physiological signals such as respiratory and cardiac signals [8]. Recent work in the field of unimodal emotion recognition agrees that building a model that can better capture the context and sequential nature of the input can significantly improve performance in the difficult task of emotion recognition. It has been shown that using a recurrent neural network-based classifier that can learn to create a more informative latent representation of the target as a whole significantly improves final performance. Based on this assumption, a deep recurrent neural network architecture was proposed to detect discrete emotions in a tweet dataset [19]. An interaction-aware attention network (IAAN) that incorporates contextual information into the learned voice representation through an attentional mechanism was proposed by Sung-Lin et al. [20]. The performance shows significant improvement over previously shown state-of-the-art and baseline methods and provides one of the best emotion recognition results [20].

### 2.2. Multimodal Approaches

As human beings, we usually rely on multiple factors such as intonation (speech), facial expression (visual modality), and contextual meaning of words (text) to detect emotions. For this reason, it is undeniably naive to expect unimodal models to outperform humans in emotion recognition and sentiment analysis. To be truly successful in emotion recognition, it is important to consider all possible mixtures of modalities. Multimodal emotion recognition is a field with many ideas and approaches and, in this part, we will focus on blending the modalities of speech, text and video. Multimodal emotion recognition has been studied using classifiers such as Support Vector Machines (SVMs) and linear and logistic regressions [21,22]. With the development of larger datasets, deep learning architectures have been developed and explored [23,24,25,26].

Shenoy and Sardana proposed context-aware emotion recognition that captures context across all modalities, bridging the gap in using the context of different inputs by using a recurrent neural network [9]. Although fusion mechanism is a popular approach in multimodal analysis, there are still some exceptions in using fusion. Features from different modalities were trained individually based on multiple classifiers. Emotion features are fused using beam search fusion learning from the beam search method [27]. In one of the recent works, instead of independently fusing the knowledge from different modalities, the attention mechanism was introduced to combine the information to perform emotion classification [10].

Pan, Zexu et al. [28] proposed a multimodal attention network (MMAN) that makes use of visual and textual signals in speech emotion recognition. Their experiment showed that identifying speech emotions profits immensely from visual and textual signals.

Siriwardhana et al. [29] used the pre-trained “BERT-like” architecture for self-supervised learning (SSL) to represent language and text modalities to learn language emotions. Their method showed that a shallow-fusion simplifies the overall structure and strengthens complex fusion mechanisms. Liu, Gaojun et al. [30] introduced a multimodal music emotion grouping approach based on music audio and lyrics. They used the LSTM network for audio modality and Bert for lyrics to describe the emotions of lyrics, which essentially addresses long-term dependency. The neural network is implemented based on linear weighted decision-making stage fusion, which increases efficiency.

### 2.3. Emotion Recognition from Art

Yanulevskaya et al. [16] proposed an approach to categorize emotions from art paintings based on an aggregation of local image statistics and SVM. Machajdik et al. [31] presented a unified framework for classifying artworks by combining low-level visual features with high-level concepts from psychology and art theory. The paper by Yanulevskaya et al. [32] introduced a “bag-of-visual-words” model combined with SVM to classify abstract paintings into positive or negative emotions. Sartori et al. [33] introduced a general learning method for emotion recognition in abstract paintings that integrates both visual and textual information.

For various reasons, most work on emotion recognition in art paintings is unimodal. Using information from different modalities could increase the model accuracy in emotion recognition. In this work, we propose a co-attention-based multimodal emotion recognition approach that aims to use information from the painting, title, and emotion category channels via weighted fusion to achieve more robust and accurate recognition.

## 3. The Proposed Sequential Multimodal Fusion Model

**Figure 1:**
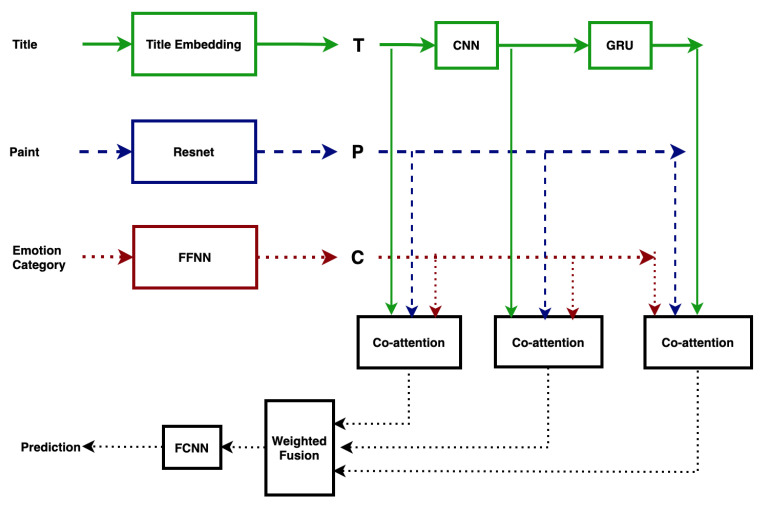
The proposed attention-based model.

Figure 1 shows the architecture of our sequential attention-based multimodal model with weighted fusion approach. Here, the title of the paint, the paint (image) and the emotion category attributes are treated as the three modalities. The weighted modality fusion technique is used to fully utilize the three modalities, and it has been shown that the model performance can be enhanced by adding the high-level concept [3,34]. In the following, the text vector, the image vector and the emotion category vector are defined and the weighted fusion technique is briefly introduced.

For the imaging modality, the pre-trained and fine-tuned ResNet [35] model is used to obtain 14×14 regional vectors of the art image, defined as the raw image vectors averaged to obtain the image vector. A Convolutional Neural Network (CNN) and a Bi-directional Gated Recurrent Unit (Bi-GRU) is used to obtain the text vectors. The word-level and n-gram level text vectors are processed using Bi-GRU to obtain the title level text feature vector. We used a three-layer feedforward neural network to obtain the emotion category feature vectors from the emotion category attributes.

To use multimodal information from all modalities and to refine the representation of all modalities, we proposed to use a sequential-based attention layer [36,37] that learns a new refined weighted representation for each of the input modalities. The refined vectors of the three modalities are combined in the modality fusion process to form a vector with weighted modality fusion [2,34] instead of simple concatenation. Finally, the fused vector is transferred to a three-layer fully connected neural network to obtain a classification result. The whole framework is shown in Figure 1 and Figure 3. More details about our model can be found below.

### 3.1. Image Feature Representation

The ResNet-50 [35] model is used to obtain representations of Art images. The last fully connected (FC) layer of the pre-trained model is chopped and replaced with a new one for the sake of model fine-tuning. Following the work of [34,36], an input image *I* is re-sized to 448×448 and divided into 14×14 regions. Each region $I_{i}$(i=1,2,⋯,196) is then sent through the ResNet model to obtain a regional feature representation, a.k.a., a raw image vector. The final image feature vector (*P*) is obtained by the average of all regional image vectors.

(1) $$P=\frac{\sum_{i=1}^{Nr}ResNet\left(I_{i}\right)}{N_{r}}$$

where $ResNet(I_{i})$ is the row image vector extracted via ResNet, $N_{r}$ (set to 196 in this work) is the number of regions as in [36]. *P* is the average of all regional image vectors.

### 3.2. Text Feature Representation

The sequence of word embeddings learned from the embedding layer was passed to a 1D convolution neural network for feature extraction at different levels. The resulting feature vector was further used to be fed to a Bi-GRU network layer to learn title level feature representation. GRU was recently introduced as an alternative to the long-short term memory (LSTM) model to make each recurrent unit to adaptively capture dependencies of different time scales [38,39]. Similarly to the LSTM unit, GRU has gating units that modulate the flow of information inside the unit, but without having a separate memory cell. The updates performed at each time step t∈{1,⋯,T} in a GRU are as follows:

Forward updates:

(2) $$\vec{Z_{t}}=sigmoid\left(\vec{W_{z}}X_{t}+\vec{U_{z}}h_{t-1}\right)$$

(3) $$\vec{r_{t}}=sigmoid\left(\vec{W_{r}}X_{t}+\vec{U_{r}}h_{t-1}\right)$$

(4) $$\vec{{\hat{h}}_{t}}=tanh\left(\vec{W_{h}}X_{t}+\vec{U_{h}}\left(r_{t}⊙h_{t-1}\right)\right)$$

(5) $$\vec{h_{t}}=\left(1-\vec{Z_{t}}\right)⊙\vec{h_{t-1}}+\vec{Z_{t}}⊙\vec{\hat{h_{t}}}$$

Backward updates:

(6) $$\overset{\leftarrow }{Z_{t}}=sigmoid\left(\overset{\leftarrow }{W_{z}}X_{t}+\overset{\leftarrow }{U_{z}}h_{t-1}\right)$$

(7) $$\overset{\leftarrow }{r_{t}}=sigmoid\left(\overset{\leftarrow }{W_{r}}X_{t}+\overset{\leftarrow }{U_{r}}h_{t-1}\right)$$

(8) $$\overset{\leftarrow }{{\hat{h}}_{t}}=tanh\left(\overset{\leftarrow }{W_{h}}X_{t}+\overset{\leftarrow }{U_{h}}\left(r_{t}⊙h_{t-1}\right)\right)$$

(9) $$\overset{\leftarrow }{h_{t}}=\left(1-\overset{\leftarrow }{Z_{t}}\right)⊙\overset{\leftarrow }{h_{t-1}}+\overset{\leftarrow }{Z_{t}}⊙\overset{\leftarrow }{{\hat{h}}_{t}}$$

where $W_{z},W_{r},W_{h},U_{r},U_{z},U_{h},U_{o}$ are the weight matrices, ⊙ is an element-wise multiplication. The activation $h_{t}$ at time *t* is a linear interpolation between the previous activation $h_{t-1}$ and the candidate activation $\hat{h_{t}}$. An update gate $Z_{t}$ decides how much the unit updates its activation or content. The reset gate $\left(r_{t}\right)$ is used to control access to the previous state $h_{t-1}$ and compute a proposed update $\hat{h_{t}}$. When off ($r_{t}$ close to 0), the reset gate effectively makes the unit act as if it is reading the first symbol of an input sequence, allowing it to forget the previously computed state.

First, the one-hot vectors of title words $T=[t_{1}\cdots t_{n}]$ are embedded individually to word level feature vectors $T^{w}=\left[t_{1}^{w}\cdots t_{n}^{w}\right]$. To compute the n-gram level features, as in [37], we applied 1D convolution on the word embedding vectors. For the *n*th word, the convolution output with window size *s* is given by

(10) $${\hat{t}}_{s,n}^{p}=tanh\left(W_{c}^{s}t_{n:n+s-1}^{w}\right),s\in 1,2$$

where $W_{c}^{s}$ is the weight parameter. Max pooling was applied to obtain the final phrase level features. Then, the final phrase level features were encoded by Bi-GRU to obtain the title level feature representation $T^{t}=\left[{\hat{t}}_{1}^{p}\cdots {\hat{t}}_{n}^{p}\right]$.

### 3.3. Emotion Category Feature Representation

**Figure 2:**
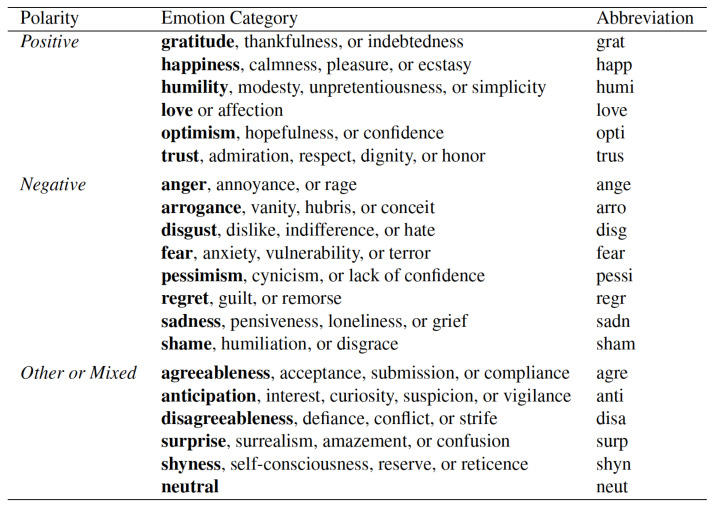
The list of emotions provided to annotators to label the title and art [1].

When the data was collected, the final class was determined using the percentage of items that were predominantly labeled with a given emotion. The list of emotion categories is shown in Figure 2. As the data was provided with the percentage of each 20-emotion category, we considered them as an input in the training process. We used a three layer feed forward neural network to learn the feature vector from the emotion category *C*.

### 3.4. Co-Attention Layer

In the co-attention layer, attention mechanism we sequentially alternate between the generation of image, title, and category attentions consisting, briefly, of five steps. Starting from the encoded title/image/emotion category features, the proposed co-attention approach sequentially generates attention weights for each feature type, using the other two modalities as guides.

Specifically, we define an attention operation [36,37] $\tilde{x}=A\left(X;g_{1};g_{2}\right)$ that takes the image or title or category feature *X* and attention guidance $g_{1}$ and $g_{1}$ derived from title and image; title and category; or category and title as inputs and outputs the attended image, title or category vector. The operation can be expressed in the following steps:

(11) $$\begin{array}{cc} H_{i} & =tanh(W_{x}x_{i}+W_{g1}g_{1}+W_{g2}g_{2}\\ \; & a_{i}=softmax\left(w^{T}H_{i}\right),i=1\cdots N\\ \; & \tilde{x}=\sum_{i=n}^{N}a_{i}x_{i} \end{array}$$

where $X=\left[x_{i};\cdots ;x_{N}\right]\in R^{d\times N}$ is the input sequence, and the fixed-length vectors $g_{1},g_{2}\in R^{d}$ are attention guidance. $W_{x},W_{g1},W_{g2}\in R^{h\times d}$ and $w\in R^{h}$ are the embedding parameters to be learned. *a* is the attention weights of the input feature *X* and the weighted sum ~x is the weighted feature [36].

In the proposed sequential co-attention approach, the encoded title/category/image features are sequentially fed as input sequences to the attention module and the weighted features from the previous two steps are used as guidance [34,36,37]. First, the title features are summarized without guidance $\left(\tilde{t_{0}}=Atten\left(T;0;0\right)\right)$ and secondly, the category features are weighted based on the summarized title features $\left(\tilde{c_{0}}=Atten\left(C;\tilde{t_{0}};0\right)\right)$.

After that, the weighted image features will be computed using the weighted emotion category features ($\tilde{c_{0}}$) and the title features $\tilde{t_{0}}$ as guidance $\left(\tilde{p}=Atten\left(P;\tilde{t_{0}};\tilde{c_{0}}\right)\right)$. In step 4 $\left(\tilde{t}=Atten\left(T;\tilde{p};\tilde{c_{0}}\right)\right)$ and step 5 $\left(\tilde{c}=Atten\left(C;\tilde{p};\tilde{t}\right)\right)$, the title and category features will also be re-weighted based on the results of the previous steps [36]. Finally, the weighted title/category/image features $\left(\tilde{t},\tilde{c},\tilde{p}\right)$ are further used for emotion prediction.

### 3.5. Weighted Modality Fusion

**Figure 3:**
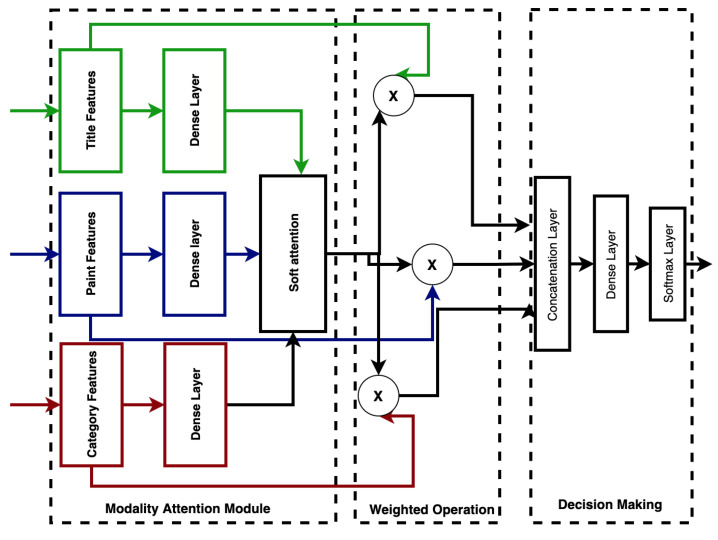
Weighted modality fusion.

Decision-level fusion is a commonly used strategy for fusing heterogeneous inputs by combining the independent modality outputs using several specific rules [34]. However, the lack of mutual association learning across modalities is a major limitation in the application of decision-level fusion [40]. We used modality attention fusion, which enables feature-level system fusion and applies weighted modality scores across the extracted features to indicate the importance of different modalities. This preserves the advantages of both feature-level fusion and decision-level fusion [40]. The feature vector for each modality is first transformed into a fixed-length form. A three-layer feed-forward neural network (FFNN) was used to compute the attention weights for each modality, which were then used in the weighted average of the transformed feature vectors, as shown in Figure 3. The result is a single vector of fixed length.

First, we implemented a three-layer feed-forward neural network to fuse the modality-specific features, and then we used softmax to generate the weighted score (s) for the given modality as follows:

(12) $$\begin{array}{cc} f & =tanh(W_{f}\left[V_{t},V_{p},V_{c}\right]+b_{f}))\\ \; & s=softmax(f) \end{array}$$

where $W_{f}$ and b+f are the trainable fusion parameters, *s* is an n-dimensional vector, and n=3 in this experiment (representing the modalities title, paint and category, respectively). We computed soft attention over the original modality features and concatenated them as in [34,40]. A dense layer was used to learn the associations over weighted modality-specific features by:

(13) $$r=tanh(W_{r}\left[s_{t}\tilde{t},s_{p}\tilde{p},s_{c}\tilde{c}\right])$$

where *r* is the final fused representation, and $W_{r}$ and $b_{r}$ are the additional parameters for the final dense layer. We made the final decision by a softmax classifier using *r* as input.

### 3.6. Classification Layer

A three-layer fully connected neural network is used as the classification layer. The activation function of the hidden layer and the output layer are Relu and softmax functions, respectively. The loss function used is the categorical cross-entropy.

## 4. Experiment and Results

### 4.1. Dataset

Mohammad and Kiritchenko [1] created the WikiArt Emotions Dataset which includes emotion annotations for more than 4000 pieces of art from four Western styles (modern art, post-Renaissance art, Renaissance Art and Contemporary Art) and 22 style categories. The art is annotated via crowd sourcing for one or more of the twenty emotion categories. The final result of closely related emotion sets were arranged in three sets, such that “positive”, “negative” and “mixed or other”, as shown in Table 1.

### 4.2. Training Details

We implemented our proposed approach in Keras using the Tensorflow backend. The pre-trained ResNet model available in Keras is used for images, and the Glove word embedding program [41] for text was used to extract row feature vectors. The parameters of the pre-trained ResNet model and the parameters of the word embeddings were set during training. The Adam optimizer was used to optimize the loss function. The best hyper-parameters are listed in Table 2. In total, 70% of the data were used as the training set, 10% as the validation set and 20% as the test set.

### 4.3. Baselines

- Bi-LSTM (Text Only): Bi-LSTM is one of the most popular methods for addressing many text classification problems. It leverages a bidirectional LSTM network for learning text representations and then uses a classification layer to make a prediction.
- CNN (Image Only): CNN with six hidden layers was implemented. The first two convolutional layers contain 32 kernels of size 3×3 and the second two convolutional layers have 64 kernels of size 3×3. The second and fourth convolutional layers are interleaved with max-pooling layers of dimension 2×2 with a dropout of 0.3. Then, a fully connected layer with 256 neurons and a dropout of 0.4 is followed.
- Multimodal approaches (text and image): two multimodal approaches, namely Resnet_GRU without attention and Resnet_GRU attention from the previous work [3], in the same task were also implemented.

### 4.4. Results and Discussion

The proposed approach was compared with the three unimodal baseline approaches and three multimodal approaches. As shown in Table 3, the proposed model improves the unimodal-based methods, which use only a single feature type, and the multimodal models, which use only information from the image and title modalities.

The proposed approach gained 8.4%, 9% and 11.5% in terms of accuracy when compared to the unimodal text-based, emotion category and image-based networks, respectively. These significant improvements confirm the importance of extracting and using information from different modalities in human emotion recognition and analysis.

Furthermore, we compared the proposed approach that uses information from the three modalities with two multimodal approaches that use information from image and title modalities, namely Resnet_GRU without attention and Resnet_GRU without attention. Our proposed approach has outperformed Resnet_GRU without attention by 6% and Resnet_GRU with attention by 3.2%.

Our proposed approach uses information from the three modalities which are image, title, and emotion category, but emotion category values are used during the training phase only. The main reason for using the emotion category during the training as one of the modality inputs is to help the model learn from expert knowledge and to see the impact of expert knowledge on model training. The experimental results have shown that using expert knowledge helped the model learn better, as shown in Table 3.

**Figure 4:**
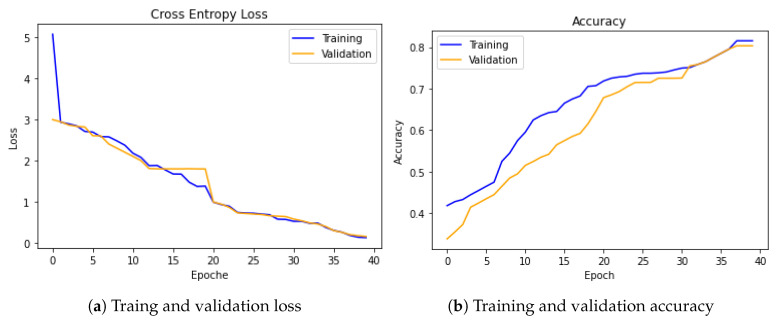
Cross-entropy loss and accuracy during the training and validation steps are shown in (**a**,**b**), respectively.

To show the advantage of weighted modality fusion over other fusion methods, we compared the weighted modality fusion model with other fusion methods. The experimental results showed that the proposed sequential-based co-attention feature learning and weighted modality fusion approaches can learn better for different categories, which implies that using pre-trained models with sequential attention and weighted modality fusion is a reasonable choice for emotion recognition from art. Figure 4 shows how our model learns from the training dataset and the generalizability of our model on the validation set, which confirms that the chosen model perfectly fits to address the emotion recognition tasks.

## 5. Conclusions

In this work, we proposed sequential-based attention to extract features from three modalities (title, art, and emotion category) and a weighted fusion approach to fuse the three modalities in the decision process. Our system used feature attention (sequential co-attention) and modality attention (weighted fusion) to select the representative information at both feature and modality levels. The experimental results on the WikiArt dataset demonstrated the effectiveness of the proposed model and the usefulness of the three modalities. Although our model was evaluated for emotion recognition in art, it can potentially be applied to other similar tasks involving different modalities.

## Figures and Tables

**Table 1:** Main characteristics of the dataset used in the experiment.

Polarity	Emotion Category	Instances
Positive	gratitude, happiness, humility, love, optimism, trust	2578
Negative	anger, arrogance, disgust, fear, pessimism, regret, sadness, shame	838
Other or Mixed	agreeableness, anticipation, disagreeableness, surprise, shyness, neutral	689

**Table 2:** The best performing hyper-parameters used for the neural networks were determined by using a grid search [3].

Hyper-Parameters	Values
ResNet FC size	512
Batch size	32
Number of BGRU hidden units	128
Dropout rate for GRU	0.4
Number of epochs	40
Learning rate	0.001
Word embedding dimensions	100

**Table 3:** Performance on test set in terms of the accuracy on the three polarities.

Model	Channel	Accuracy	Loss
CNN	Image	0.683	0.663
Bi-LSTM	Title	0.658	0.810
FFNN	Category	0.689	0.441
ResNet_GRU without attention	Paint, title	0.713	0.710
ResNet_GRU with attention	Paint, title	0.741	0.130
Our new model with concatenation	Paint, title and category	0.724	0.684
Our new model	Paint, title and category	0.773	0.143

## Data Availability

The data presented in this study are openly available at WikiArt Emotions Project webpage: http://saifmohammad.com/WebPages/wikiartemotions.html (accessed on 16 August 2021).
